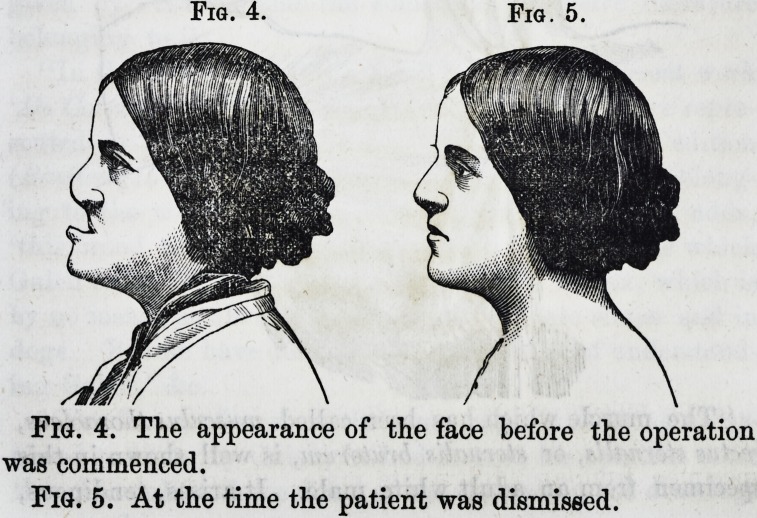# Deformity Occasioned by Contraction of the Arch of the Superior Maxillary and Irregularity of the Teeth, Successfully Treated

**Published:** 1858-01

**Authors:** W. Dalrymple

**Affiliations:** Dentist, New York.


### ARTICLE XII.

*Deformity occasioned by Contraction of the Arch of the Superior Maxillary and Irregularity of the Teeth, successfully Treated.* By DR. W. DALRYMPLE, Dentist, New York.

EDITORS OF THE AM. JOUR. OF DENTAL SCIENCE:

*Dear Sirs*—Through the kind appreciation of Stephen Smith, M. D., editor of the New York Journal of Medicine, and at his solicitation, I was induced to take professional charge of a case presenting many peculiarities with extraordinary obstacles to be overcome. I have had the same under treatment for something over a year with a view to correct a deformity arising from very great contraction of the arch of the upper jaw, and involving also irregularities of the teeth. As doubts have been expressed by some of our best surgeons as to the possibility of relief through dental appliances I am induced to send you a history of the case and the means by which the deformity was remedied.

The patient was a very interesting girl, 13 years of age, who had been operated upon in infancy for double hair lip. But the operation had only partially relieved the deformity. A large portion of the alveolar process with the gums of the deciduous incisor teeth were removed by the operation, and at the time I saw her there was nothing left between the canines but two elemental portions of teeth, which I subsequently removed. The articulation of the teeth was such as to leave the remaining upper teeth in their entire circle, closing on the inside of the lower ones. The upper ones being firmly clasped by the teeth of the lower jaw, and of course so acting upon them as constantly to increase and confirm the deformity. The superior cuspid teeth were brought almost together.

The palatine arch was so contracted that the sides on perpendicular portions were brought in positive contact, so much so that it was difficult to pass a thin letter folder into the fissure thus formed. The lower jaw was protruding to a very great extent—the superior canines closing about one-fourth of an inch inside and back of the lower ones. It was very evident that the action of the lower teeth upon the upper ones must within a short time have brought the upper canines and bicuspids quite together. Add to this the contraction of the upper lip which was greatly shortened from being cut away and from having no teeth to support it—and the fact that the cartilage or point of the nose was drawn down, and you have a portrait of the case as I found it.

Having many doubts of the practicability of any operation to overcome these manifold difficulties I gave but little encouragement to patient or friends. Yet being confident that the only remedy must come from our profession, I was willing to make an attempt. My first object, so to speak, was to unlock the jaws; and to break up the constant tendency by shutting the mouth, to increase the difficulty. This was done by first preparing a firm plate for the basis of my future movements, upon which was soldered studs to keep the jaws apart, to break up the articulation, and at the same time so arranged as to act as inclined planes in moving the two sides of the upper jaw outwards or from each other whenever the jaws were closed.

This fixture was placed on the lower teeth, and was worn for some weeks, but not making any considerable progress, I was induced, in connection with it, to try the bandage described by Dr. A. Westcott.* This was worn only during the night, as then the muscles being in a relaxed state, and no voluntary resistance to its action would be exerted by the patient, the effect was doubtless nearly as great as if worn during the whole twenty-four hours.

With the view of accomplishing the same end—that of spreading the jaws laterally, I also used a bar of wood reaching from one canine tooth to the other, and of such length as to press firmly upon them. This was supported in its place by a plate that was swaged to fit accurately to the palatine portions of the upper jaw, and about the necks of the teeth. To this was soldered too loops through which was passed the bar or brace. These were renewed and lengthened from day to day, and were of the greatest service in producing the desired effect. I beg leave here to say, that in all cases where bars or braces are to be employed for similar purposes, I regard wood by far the best material. This is more agreeable to the sense of taste—less unpleasant in its pressure upon the tooth—is more easily adjusted and

* See Am. Jour. Dental Science, vol. 5, No. 2, page 147.

retains its position far better than any metallic substance. I would here take occasion to speak of the very ingenious "little jack screw," of Dr. Dwinelle, a most effectual apparatus in many cases, yet in the present I was wholly unable to use it, owing to excessive galvanic irritation. This, however, may be in part owing to the manner in which I applied the fixture, as Dr. D. informs me that he has applied these fixtures in precisely similar cases, without the least galvanic irritation. Indeed, I believe he claims for the lifting screws, that, notwithstanding they are constructed of steel, while they are loaded with zinc the latent galvanic action thus evolved forbids their rusting or consequent irritation. The combined operation of the inclined planes, bandage and wooden bars had the effect within four or five months, to spread the jaws sufficiently to bring the upper bicuspid and molars of this jaw to fairly antagonize with the corresponding ones of the lower jaw, and also to greatly diminish the protrusion of the chin. While this was advancing, I was constantly bringing forward the teeth of the upper jaw by the use of wooden *wedges*. Commencing with the canine teeth, which were thus moved about one-eighth of an inch. The teeth thus brought forward were kept in their place by soldering upon the plate, (which had now to be re-swaged to fit the present state of the mouth,) collars or clasps. After having thus secured the canines in their new position, the next or first bicuspids were treated in the same way, so as to bring them in contact with the cuspidati. The first collar or clasp was now removed and a similar one placed behind the bicuspid, and so on until the entire arch was sufficiently spread in this direction. Notwithstanding I had now succeeded in correcting two important features in this deformity—that of restoring the arch of the upper jaw to its proper form and that of retracting the chin, still a third difficulty remained which bid fair to be more formidable than either of the others, I allude to the greatly contracted state of the upper lip and the depression of the nose. Had this been properly attended to in infancy, in connection with the operation for hare lip, it would have been comparatively an easy task. But at the present age of the patient, I had serious doubts about complete success. I made no attempt at restoring this portion of the deformity till the operation for correcting the irregularity of the teeth and jaws was fully completed, and the mouth was in all respects prepared to receive a plate upon which were to be attached teeth to supply the missing ones.

I at first entertained the hope that this could be accomplished by carrying the plate very high up under the lip, and by giving it proper thickness to overcome this contraction by continued pressure, but in this I was disappointed, the irritation being two great to be borne by the patient. I was under the necessity of resorting to the scalpel, and to make a pretty free separation of these contracted parts (*septum nasi*) from the jaw to which they were firmly attached. This done, I had no difficulty in preparing a plate for teeth and restorer passing much farther upwards, which gave proper form to the lip, and relieved the tension upon the point of the nose. I used for the plate thin platina, and to secure a thin and unirritating surface in contact with the parts which had been cut I returned downwards about a line in breadth, so as to secure a smooth rim, filling up the interspace and below to give it the desired shape with "Allen's body" or continuous gum. Upon introducing the piece, the tension upon it caused by the excessive pressure of the contracted lip a considerable inflammation. However it kindly healed in the course of a few days. The parts assumed in all respects a healthy condition, with each feature of a most unsightly deformity more perfectly corrected than I at first dared to hope.

FIG. 1.

FIG. 1. The appearance of the mouth and articulation of the teeth.

FIG. 2.

FIG. 2. After the operation of expanding the jaws and regulating the teeth was completed with artificial plate and teeth.

FIG. 4.

FIG. 5.

FIG. 4. The appearance of the face before the operation was commenced.
FIG. 5. At the time the patient was dismissed.

## Figures and Tables

**Fig. 1. f1:**
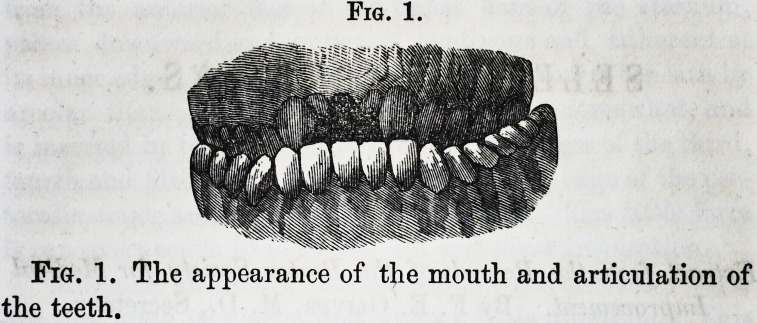


**Fig. 2. f2:**
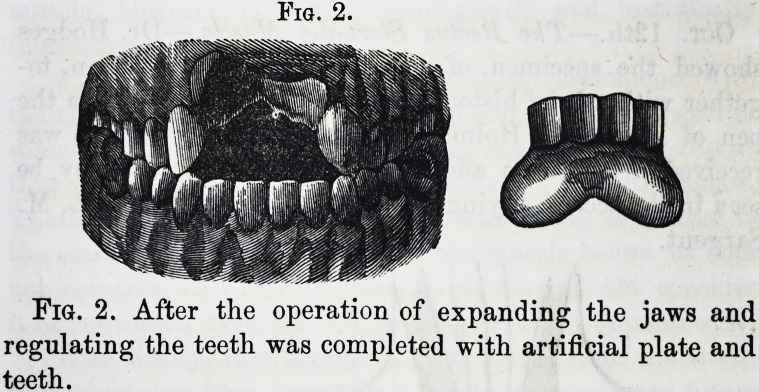


**Figure f3:**